# Cellular selectivity of AAV serotypes for gene delivery in neurons and astrocytes by neonatal intracerebroventricular injection

**DOI:** 10.1371/journal.pone.0188830

**Published:** 2017-12-15

**Authors:** Sean L. Hammond, Ashley N. Leek, Evan H. Richman, Ronald B. Tjalkens

**Affiliations:** 1 Department of Environmental and Radiological Health Sciences, Colorado State University, Fort Collins, CO, United States of America; 2 Department of Biomedical Sciences, Colorado State University, Fort Collins, CO, United States of America; University of Kansas Medical Center, UNITED STATES

## Abstract

The non-pathogenic parvovirus, adeno-associated virus (AAV), is an efficient vector for transgene expression *in vivo* and shows promise for treatment of brain disorders in clinical trials. Currently, there are more than 100 AAV serotypes identified that differ in the binding capacity of capsid proteins to specific cell surface receptors that can transduce different cell types and brain regions in the CNS. In the current study, multiple AAV serotypes expressing a GFP reporter (AAV1, AAV2/1, AAVDJ, AAV8, AAVDJ8, AAV9, AAVDJ9) were screened for their infectivity in both primary murine astrocyte and neuronal cell cultures. AAV2/1, AAVDJ8 and AAV9 were selected for further investigation of their tropism throughout different brain regions and cell types. Each AAV was administered to P0-neonatal mice via intracerebroventricular injections (ICV). Brains were then systematically analyzed for GFP expression at 3 or 6 weeks post-infection in various regions, including the olfactory bulb, striatum, cortex, hippocampus, substantia nigra (SN) and cerebellum. Cell counting data revealed that AAV2/1 infections were more prevalent in the cortical layers but penetrated to the midbrain less than AAVDJ8 and AAV9. Additionally, there were differences in the persistence of viral transgene expression amongst the three serotypes examined *in vivo* at 3 and 6 weeks post-infection. Because AAV-mediated transgene expression is of interest in neurodegenerative diseases such as Parkinson’s Disease, we examined the SN with microscopy techniques, such as CLARITY tissue transmutation, to identify AAV serotypes that resulted in optimal transgene expression in either astrocytes or dopaminergic neurons. AAVDJ8 displayed more tropism in astrocytes compared to AAV9 in the SN region. We conclude that ICV injection results in lasting expression of virally encoded transgene when using AAV vectors and that specific AAV serotypes are required to selectively deliver transgenes of interest to different brain regions in both astrocytes and neurons.

## Introduction

Adeno-associated viruses (AAVs) are the most commonly used vector for gene delivery to central nervous system (CNS). AAVs are small (20nm), non-pathogenic in humans and animals, contain a single-stranded DNA genome and are a member of the *Parvoviridae* family within the *Dependovirus* genus [1] [2]. Serology of AAVs is an important functional characteristic for cell specific transduction efficiency within the CNS. AAV2 was the first serotype cloned into a bacterial plasmid and has since been used as a comparison to identify other serotypes. Each serotype has a different CNS transduction capacity and does not cross-react with naturally-occurring human AAV2-neutralizing antibodies [3,4]. Twelve serotypes (AAV1-12) have been tested thoroughly for their ability to transduce specific cell types and tissue and differentiated between capsid protein motifs that bind specific cell surface receptors for cell attachment [4]. For example, AAV1, AAV4, AAV5, AAV7 (sialylated glycoproteins), AAV2/AAV3 (heparin sulfate proteoglycans), AAV9 (galactose) and AAV8 do not have a known primary receptor, although laminin is a potential co-receptor for these viruses [4,5,6 7]. More recently, a universal receptor, KIAA0319 or AAVR, has been identified that mediates rapid endocytosis after cell binding and attachment for all AAV serotypes [8]. AAV hybrid serotypes or pseudo-serotypes have been created by viral engineering, which are constructed with integrated genome containing (cis-acting) inverted terminal repeats (ITR) of AAV2 and capsid genes of other serotypes for increased viral specificity and transduction [9]. Several studies display AAV serotype transduction differences *in vitro* and *in vivo*; dependent on cell type specificity, cell toxicity, viral delivery method, viral delivery timing, and AAV transgene expression stability over time [9–12]. However, some of these studies lack informative, transitional data incorporating both hybrid and wild type serotype differences found in neural cell culture preceding serotype comparisons in mouse brain.

Neonatal intracerebroventricular (ICV) injection is a promising delivery technique for AAVs in mice that is minimally invasive and displays widespread tropism throughout the brain, as opposed to stereotactic injection procedures in adult mice, which are highly invasive and localize only to site of injection. Previous studies reported that when AAV2 is injected directly into the cerebral lateral ventricles at birth, it can circulate through the subarachnoid space, disseminate through the ventricle ependymal cell lining and deliver viral vector throughout the CNS [13]. Transgene expression following neonatal ICV injection can persist for at least 12 months, and there are regional differences in tropism amongst different AAV serotypes delivered by this method[14]. Because of the reported variability in serotype-dependent regional tropism amongst different studies, there remain questions as to which serotype is best suited to transduce neurons or astrocytes in specific regions of interest within the CNS by ICV [4].

In the present study we therefore used multiple AAV serotypes expressing fluorescent GFP reporters to examine regional tropism and efficiency in transducing gene expression in primary neuron and astrocyte cultures and *in vivo*. AAVs were administered by ICV injection into neonatal P0 mice for delivery to the CNS. Tissue was collected at 3 and 6 weeks post-ICV injection to compare AAV serotype stability in multiple regions of the brain. Previous investigations of AAV serotype differences have predominately focused on the localized expression after stereotaxic injection in adult mice for intervention in neurodegenerative disease models. Still, few studies have fully investigated the capacity of multiple ICV delivered AAVs serotypes to penetrate to deep ventral midbrain regions and transduce specific cell types [10,14,15]. Using immunofluorescence and imaging, we report different patterns of cell specific AAV serotype tropism in cell culture and in multiple regions of mouse brain, including the substantia nigra (SN). Further testing was conducted with glial fibrillary acidic protein (GFAP) promoter-driven AAV using the optimal serotype to exclusively target astrocytes. These studies identified different AAV serotypes that preferentially transduced gene expression in astrocytes or neurons with surprising variability in regional tropism, suggesting several suitable serotypes for achieving gene expression with the desired regional and cellular selectivity.

## Materials and methods

### AAV serotypes

The following AAV serotypes tested were acquired from Vector BioLabs; AAV1(Cat# 7002), AAV2/1(Cat# 7071), AAV2/DJ (Cat# 7078), AAV2/DJ8 (Cat# 7118), and AAV2/DJ9 (Cat# 7119) and astrocyte-specific, AAVDJ8-GFAP-mCherry-WPRE. AAV8 and AAV9 were from Virovek. All pseudo-serotypes incorporated ITRs of wildtype AAV2 and mRNA stabilizing woodchuck hepatitis virus posttranscriptional regulator elements (WPRE). AAV2/1 had capsid protein of wildtype AAV1, AAVDJ was a synthetic serotype made from 8 wildtype serotypes, AAVDJ8 was AAVDJ modified to specific residues of AAV8, and AAVDJ9 was modified to specific residues of AAV9. Each pseudo-serotype also had a CMV/Chicken-beta-actin hybrid promoter and eGFP transgene. Wildtype AAV1 had a CMV promoter and an eGFP transgene. All Vector BioLabs serotypes were stored in a PBS/glycerol 5% stock at an initial concentration of 1X10^13^ GC/ml. Serotypes acquired from Virovek had a CMV promoter, a GFP reporter and a modified wildtype capsid. AAV8 was an initial concentration of 2.14X10^13^ GC/ml and AAV9 was at 2.10X10^13^GC/ml, stored in a PBS/pluronic F-68 0.001% stock, before dilution to working concentration. All serotypes were made in aliquots to minimize freeze/thaw cycles and stored at -80°C.

### Use of animals

Timed pregnant female C57BL/6 mice were obtained E16-E19 (Charles River aged 3–4 months). P0 neonatal mice were ICV injected within ~12 hours post birth. Mice were housed in a 12hr-light/dark cycle and temperature controlled room (maintained 22–24°C) with access to standard chow and water *ad libitum*. All animal procedures were conducted in compliance with National Health Institute guidelines and approved by Colorado State University Institutional Animal and Use Committee. Neonatal ICV injections were conducted under heavy cyroanesthesia. Termination of adult animals was performed by decapitation with isoflurane anesthesia under the supervision of veterinary staff per an approved IACUC protocol.

### *In vitro* AAV transduction experiments and immunostaining

Primary cortical neurons were isolated from P0 neonatal C57BL/6 mice, as previously described, and then seeded on Poly-D-Lysine (Sigma Cat# P6507-5mg) coated 12-mm coverslips at a density of 5.0X 10^4^/well[16]. For live cell fluorescent plate reading/imaging, neurons were seeded at 5X10^3^/well in 96-well black-walled plates (Thermo Scientific, Waltham MA). Neuronal cultures were allowed to grow for 7 days prior to viral treatments. For mixed glial cell isolations, astrocytes were also isolated from P0 neonatal C57BL/6 mice, as previously described [17]. Mixed glia were seeded on FBS coated 12-mm coverslips at 5.0X 10^4^/well 24 hours prior to viral transductions. All AAV-GFP serotypes were diluted to 5X10^10^GC/ml in Neurobasal Medium (Life Technologies) for primary neurons or serum-free MEM/EBSS (Hyclone) for astrocyte transductions. Native GFP fluorescence signal was monitored at 488nm emission/519nm each day on a Cytation3 Cell Imaging Multi-Mode plate reader (BioTek Instruments, Winooski, VT). DIV 7 cells were washed with phosphate-buffered saline (1X PBS) and replaced growth medium with Fluorobrite DMEM (Life Technologies) for GFP and bright-field 20X objective imaging on plate reader, then fixed with ice cold methanol for 20 min at -20°. For mixed glia transduction experiments with AAVDJ8-GFAP-mCherry, a higher titer of 1.9X10^11^GC/ml was necessary for successful detection of mCherry expression and cells were fixed at DIV 11. Both cell types were immunostained for chicken polyclonal anti-GFP (1:500; AvesLabs Cat# 1020), rabbit polyclonal anti- mitogen associated protein (MAP2) (1:500; Abcam Cat# 32454) for neurons and mouse polyclonal anti-GFAP (1:500; Cell Signaling Cat# 3670S) for astrocytes and anti-mCherry (1:100; Abcam) for AAVDJ8-GFAP-mCherry transduced cultures. Secondary antibodies used were AlexaFluor donkey anti-rabbit 555, goat anti-chicken 488, and donkey anti-mouse 555 (1:500; Life Technologies). All 12mm-coverslips were mounted on glass slides with VectaShield mounting medium containing 4′,6-diamidino-2-phenylindole (DAPI; Vector, Burlingame, CA) and stored in 4°C until imaged.

### Neonatal Intracerebroventricular injections

ICV injection procedures were closely adapted from several established protocols by Kim et al. 2013 & 2014 and Charkrabarty et al. 2013 [5,14,18]. In brief, P0 neonatal pups were induced with hypothermic anesthesia by placement on a cold aluminum plate in ice. Anesthesia was confirmed by neonatal color change from pink to purple, squeezing of paw and cessation of movement before injections. ICV injections were performed using a 10uL Hamilton micro syringe with a 32 G, 0.5”, 30° bevel RN needle. Ventricular injection sites were identified by 2/5 distance from lambda suture to eye and 3mm ventral from skin (marked on needle shaft). Working viral solutions were diluted in PBS at 1X10^10^GC/uL, and injected as a 2uL volume/hemisphere, equivalent 2X10^10^GC/hemisphere. Injected pups were placed on warming pad and regained movement before returned to dam cage. Juvenile weanlings were terminated at 3 weeks for brain collection or weaned/aged for an additional 3 weeks for 6-week post ICV injection.

### Histological preparation of tissue and immunostaining

AAV injected mice were terminated at 3/6 weeks under deep isoflurane anesthesia and decapitated for rapid brain dissection. Dissected brains were stored in 3% paraformaldehyde overnight and then stored in cacodlyate-PBS containing 15–30% sucrose at 4°C until processed for cryo-sectioning. Brains were then frozen in OCT and sectioned at 40μm thickness (coronal/sagittal) on microtome. Free-floating brain regions of interest (ROI) were mounted on glass slides and imaged for AAV-GFP or stored in cryoprotectant at -20°C until immunostaining. Immunofluorescent tissue staining was conducted as previously described by Miller et al. [19], with the addition of antigen-retrieval by incubating tissue sections in 0.01 M sodium citrate buffer (pH 8.45) for 20 min prior to blocking. Primary antibodies diluted in 0.1% triton-X containing tris-base-saline (TBS) are rabbit polyclonal anti-MAP2 (1:500; Abcam Cat# 32454), rabbit polyclonal anti-S100β (1:100; Abcam Cat# ab41548), chicken polyclonal anti-GFP (1:500; AvesLabs Cat# 1020), and rabbit polyclonal anti-tyrosine hydroxylase (1:500; Millipore Cat#: AB152). Sections were stained for DAPI (Sigma) and mounted on glass coverslips in VectaShield mounting medium and stored at 4°C until imaging.

### CLARITY tissue-transmutation

Passive clarification was conducted similarly to protocol established by Tomer et al. 2014 [20]. In brief, brains were embedded in hydrogel (4% acrylamide/0.05% Bis-acrylamide) and sectioned at 400um thickness on a cryo-microtome. Sections were placed in clearing solution (4% sodium dodecyl sulfate/200mM boric acid, pH 8.5) for 7 days at 37°C and 35 rpm. Sections of clarified substantia nigra region were then selected for immunostaining. Clearing solution was removed by washing 3 times with TBS and then sections were incubated for immunostaining with chicken polyclonal anti-tyrosine hydroxylase (1:200; Abcam Cat# ab76442), rabbit polyclonal anti-GFAP (1:200; DAKO Cat# Z0334), anti-GFP (1:200; AvesLabs) or anti-mCherry (1:200; Abcam Cat# ab167453). Antibodies were diluted in TBS at 37°C and incubated with tissue sections with an orbital shaker at 35 rpm overnight and then at 4°C for 1 day afterward. Multiple washes were performed over 2 days at 37°C (with orbital shaking at 35 rpm) and then cleared sections were placed in TBS containing goat anti-chicken AlexaFluor 647 (1:200; Life Technologies) and donkey anti-rabbit AlexaFluor 555 (1:200; Life Technologies) at 37°C overnight with orbital shaking (35 rpm). The next day, sections were washed several times with TBS and stored at 4°C until imaged.

### Imaging and cell counting

Images of transduced primary cells and all quantitated AAV infected brain regions were acquired using a 20X air objective with a Zeiss Axiovert 200M inverted fluorescent microscope equipped and a Hammatsu ORCA-ER-cooled charge coupled device camera (Hammatsu Pho-tonics, Hammatsu City, Japan). For IF quantitation, multiple random, z-stack images of approximately 30 μm dissector height for both hemispheres of all brain regions (*in vivo* quantitation) or a single 2D images (for *in vitro* quantitation) were acquired per coverslip/brain region and counted total %GFP cell/image field using Slidebook software (version 5.5, Intelligent Imaging Innovations, Denver CO). A background subtraction was performed for all IF *in vivo* images, prior to quantitation. Representative high-magnification images were acquired with a Zeiss Plan-Apochromat 100X oil objective lens. Representative 10X objective montage images of ROI were acquired with a BX51 microscope (Olympus, Center Valley, PA, USA) equipped with a Hammatsu ORCA-Flash4.0 digital CMOS camera, ProScan III stage controller (Prior, Rockland, MA USA) and CellSens Dimension software (version 1.12, Olympus, Center Valley, PA, USA). All bright field images of mock-injected brains were acquired with Olympus SZX12 stereo-dissecting microscope. For enhanced CLARITY fluorescence imaging of AAVDJ8-GFP, 40X oil objective montage images were acquired using a Fluoview 1200 scanning-laser confocal microscope to penetrate ~80μm thickness of tissue (Olympus, Center Valley, PA USA). CLARITY fluorescent imaging of AAVDJ8-GFAP-mCherry was conducted on a Zeiss LSM 510 Laser-scanning confocal microscope to penetrate and capture ~180μm thickness of tissue. Both sets of CLARITY images were 3D-rendered in ImageJ analysis software [21]. Representative images of whole brain fluorescence were acquired with ChemiDoc MP imaging system (BioRad). Uninjected controls were imaged with each serotype for comparison of background fluorescence (**Fig C in S1 File)**.

### Statistical analysis

All data are expressed as mean ± SEM. Grouped analyses was performed using a Two-way ANOVA with Sidak’s post hoc test to compare biological replicate means between groups. For semi-quantitative representation of GFP^+^ cells/brain region as depicted in **Table 1,** cell counts were divided into quartiles as follows; +++≤55.5 GFP^+^ cells/image field, ++≤31.3 GFP^+^ cells/image field, +≤11.6 GFP^+^ cells/image field and -≤5.8 GFP^+^ cells/image field. Statistical significance was identified as *p <0.05, **p <0.01, ***p <0.001, ****p <0.0001. All statistical analyses were conducted using Prism (version 6.0; Graph Pad Software, San Diego, CA).

**Table 1:** Semi-quantitative assessment of AAV serotype tropism in mouse brain.

	3 Weeks Post-Injection			6 Weeks Post-Injection		
Serotype	AAV2/1	AAVDJ8	AAV9	AAV2/1	AAVDJ8	AAV9
**cortex**	+++	+++	+++	++	+++	+
**cerebellum**	++	-/+	+	-/+	-/+	-/+
**hippocampus**	+++	+++	++	++	+++	-/+
**olfactory bulb**	+++	++	+	+++	++	-/+
**striatum**	+	+	+	+	++	-/+
**substantia nigra**	-/+	+	++	-/+	++	+

## Results

### Comparison of multiple AAV serotypes in primary astrocyte and neuronal cultures.

**Fig 1:**
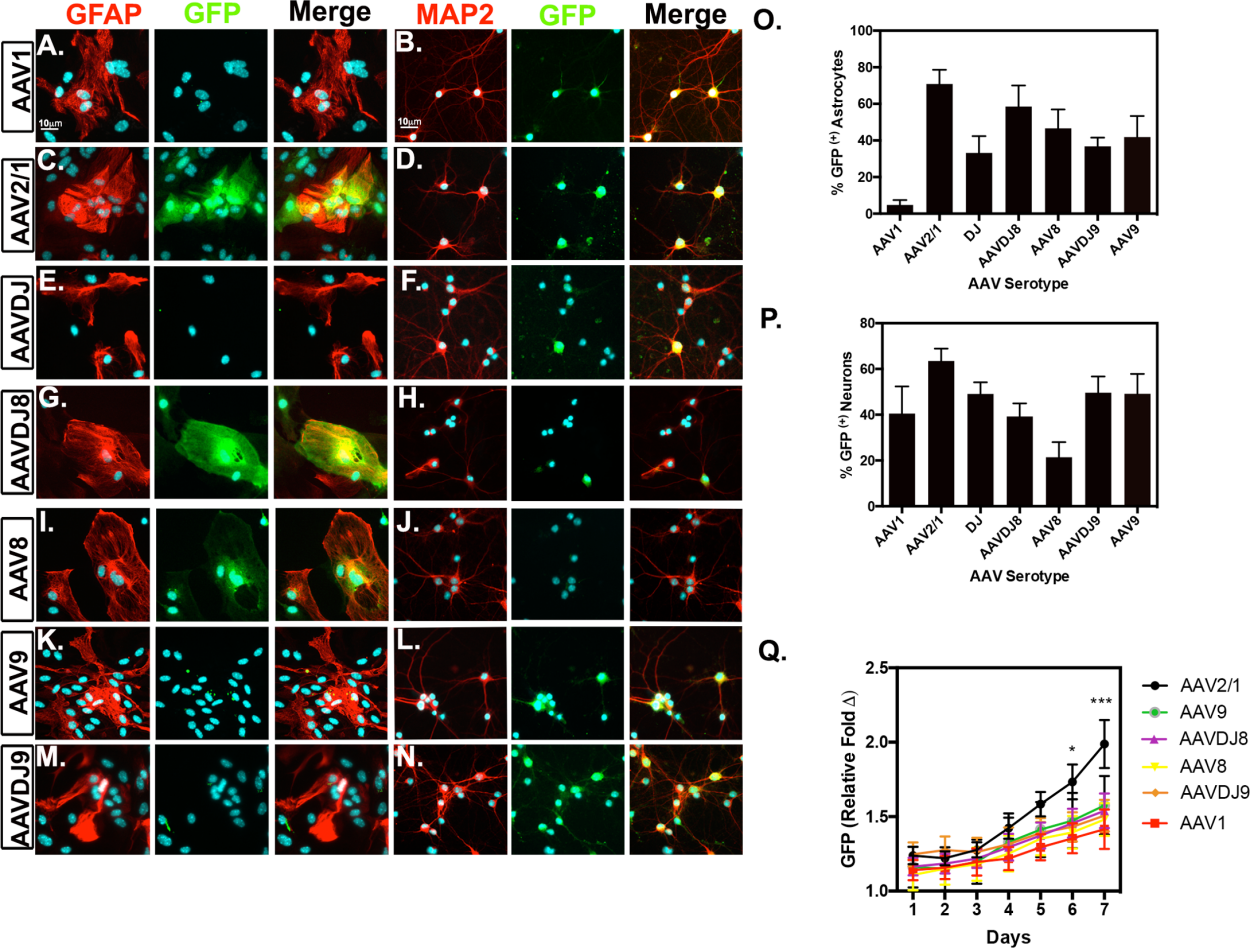
AAV2/1 is most efficient in primary neuron and astrocyte cultures. Primary neuron and astrocyte cultures transduced with multiple AAV serotypes for comparison by immunofluorescent analysis with astrocyte marker, GFAP (red; left) and neuronal marker MAP2 (red; right), GFP (green) and DAPI (cyan). Representative images of virally transduced GFP reporter are depicted in 20X objective images of (**A-B**) AAV1, (**C-D**) AAV2/1, (**E-F**) AAVDJ, (**G-H**) AAVDJ8, (**I-J**) AAV8, (**K-L**) AAV9, (**M-N**) and AAVDJ9. Percent of GFP^+^ astrocytes (**O**) and (**P**) neurons were quantitated by co-localization with specific cell marker. (**Q**) Live cell fluorescence was measured in primary neuronal cultures by native GFP detection each day for 7 DIV (*n* = 3-7/serotype; fixed astrocytes, *n* = 6-10/serotype; fixed neurons, *n* = 6/serotype; live cell; representation of three separate experiments; *p<0.05, ***p<0.001).

Several wildtype and hybrid serotypes carrying eGFP or GFP fluorescent reporters were compared for transduction efficiencies in astrocytes. Mixed glia cultures were transduced for 1 week with different serotypes and fixed for double immunofluorescent labeling for the astrocyte marker, GFAP (red) and GFP (green) (**Fig 1A, 1C, 1E, 1G, 1I, 1K and 1M)**. Astrocyte (GFP^+^GFAP^+^/GFAP) transduction efficiencies (Mean ± SEM; *n* = 3-7/serotype) were as follows: 4.785%±2.635 for AAV1, ~71%±7.79 for AAV2/1, 33.17%±9.169 for AAVDJ, 58.49% ±11.43 for AAVDJ8, 46.64%±10.31 for AAV8, 36.77%±4.749 for AAVDJ9, and 41.88%±11.49 for AAV9 (**Fig 1O)**. Similarly, primary cortical cultures were transduced for 1 week and fixed for double immunofluorescent labeling for the neuronal marker, MAP2 (red) and GFP (green) (**Fig 1B, 1D, 1F, 1H, 1J, 1L and 1N)**. Neuron (GFP^+^MAP2^+^/MAP2^+^; *n* = 6-10/serotype) transduction efficiencies were quantitated as follows: 40.5%±11.9% for AAV1, 63.5±5.4% for AAV2/1, 49.1±5.0% for AAVDJ, 39.2±5.7% for AAVDJ8, 21.4±6.6% for AAV8, 49.6±7.1% for AAVDJ9, 49.2±8.7% for AAV9 (**Fig 1P)**. AAV-GFP fluorescence intensity was also monitored in neuronal cultures once per day for 1 week via live cell imaging using a fluorescence microtiter plate reader. AAV-GFP expression was noticeable at approximately 3 days post-infection and increased every day *in vitro* (DIV). AAV2/1 displayed a significantly higher fold-change in fluorescence intensity compared to all other serotypes at 6 DIV (1.733±0.117) and 7 DIV (1.988±0.161) (*n* = 6/serotype/time point; *p<0.05, ***p<0.001) (**Fig 1Q**).

### Intracerebroventricular injection of AAV constructs

**Fig 2:**
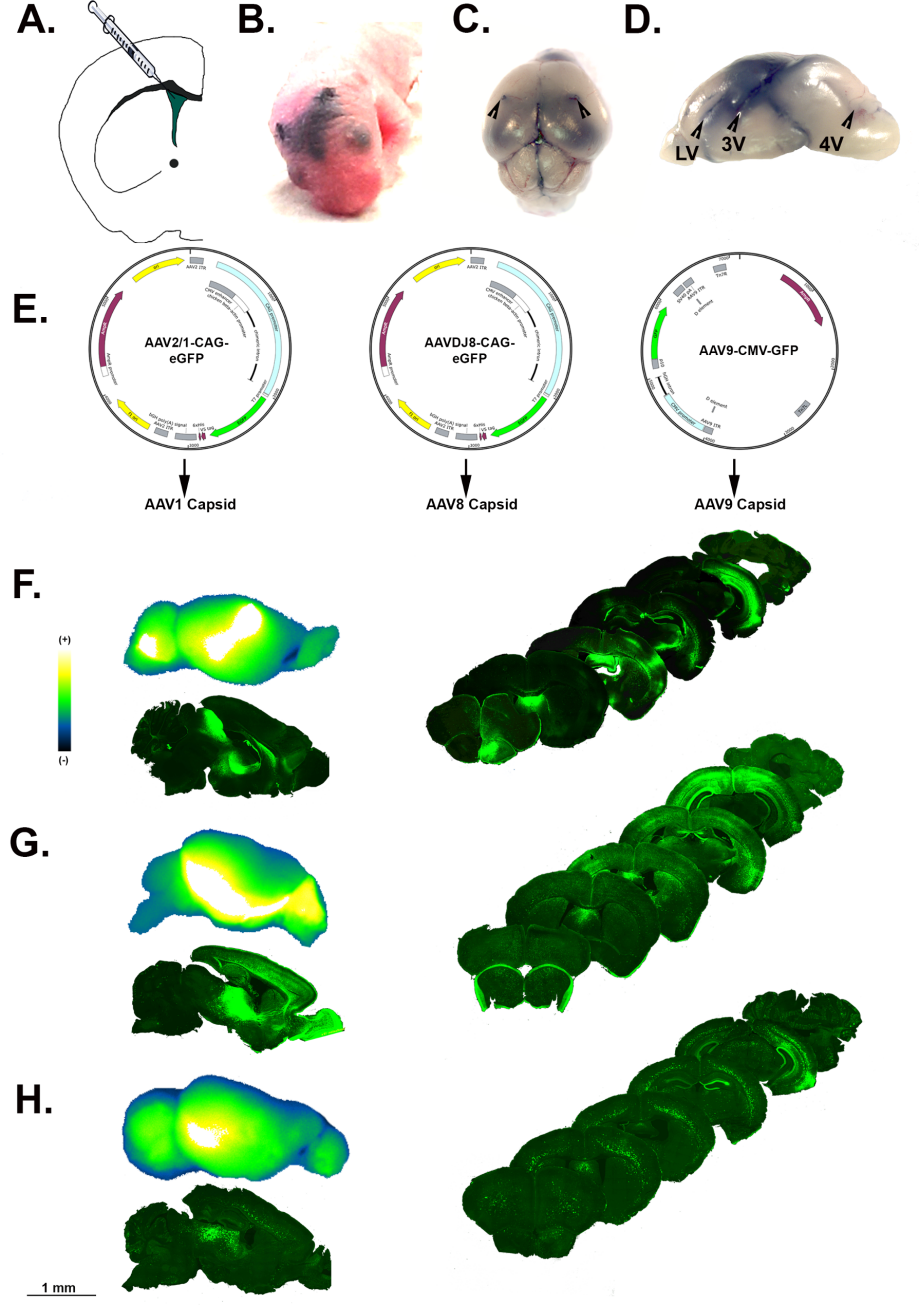
Intracerebroventricular injection of multiple AAV serotypes in neonatal P0 mice. (**A**) P0 neonatal mice were injected directly into the lateral ventricles for widespread viral solution as illustrated in cartoon schematic. (**B**) Mock injection of trypan blue/PBS solution was used to test successful target location at 2/5 from bregma suture and eye landmark. (**C**) Black arrows identify injection sites on dissected whole brain and (**D**) lateral ventricles (LV), 3^rd^ ventricle (3V) and 4^th^ ventricle (4V) on gross incised sagittal brain. (**E**) Plasmid AAV (pAAV) vector maps identify specific promoter/reporter elements of selected serotypes AAV2/1, AAVDJ8 and AAV9. Lateral plane view pseudo-colored whole brain native GFP fluorescence of (top), medial view of 10X objective montage images for sagittal cross sections (bottom) and rostral-caudal coronal cross sections (right) of (**F**) AAV2/1, (**G**) AAVDJ8, and (**H**) AAV9 injected brains at 3-weeks post injection (pseuodocoloring scale of fluorescence: white = highest, black = lowest; all images are representation of 3–4 animals/serotype).

To administer several selected AAV serotypes to the murine CNS, ICV injections were conducted on P0 neonatal mice (**Fig 2**). Lateral ventricles were targeted for injection on both hemispheres as depicted in the schematic in **Fig 2A**. Successful injection into ventricle space was confirmed by mock injection of trypan blue/PBS solution administered to neonatal P0 mouse brain for visualization of injection distribution and imaged on bright field stereo dissecting microscope, arrowheads indicate injection sites (**Fig 2B and 2C**). Mock injected brains were incised along coronal and saggital planes to visualize sites of viral solution spread from the lateral ventricles (LV), third (3V), mesencephalic aqueduct (MA) and fourth ventricle (4V), arrowheads indicate ventricular spaces (**Fig 2D, Fig A in S1 File**). For *in vivo* AAV serotype characterizations, AAV2/1, AAVDJ8 and AAV9 were selected based on the capacity to efficiently transduce primary neural cultures and the noticeable distribution differences of other serotypes by ICV injection (**Fig 1, Fig D in S1 File**). Basic plasmid (pAAV) maps of each serotype are illustrated in **Fig 2E**. To monitor AAV tropism throughout the CNS, whole brains were ICV injected with AAV2/1 (**Fig 2F**), AAVDJ8 (**Fig 2G**) and AAV9 (**Fig 2H**) and dissected 3 weeks post-injection or imaging of intrinsic GFP expression. Lateral views of each whole brain are shown in the top image in pseudo color (**Fig 2F–2H**). Medial intrinsic fluorescence is depicted in representative images of sagittal cross sections (bottom), along with serial coronal cross sections rostral to caudal on right of each panel, depicting penetrance of each AAV serotype throughout the CNS (**Fig 2F–2H**).

### Quantitation of AAV2/1-GFP tropism at 3 and 6 weeks post-injection

**Fig 3:**
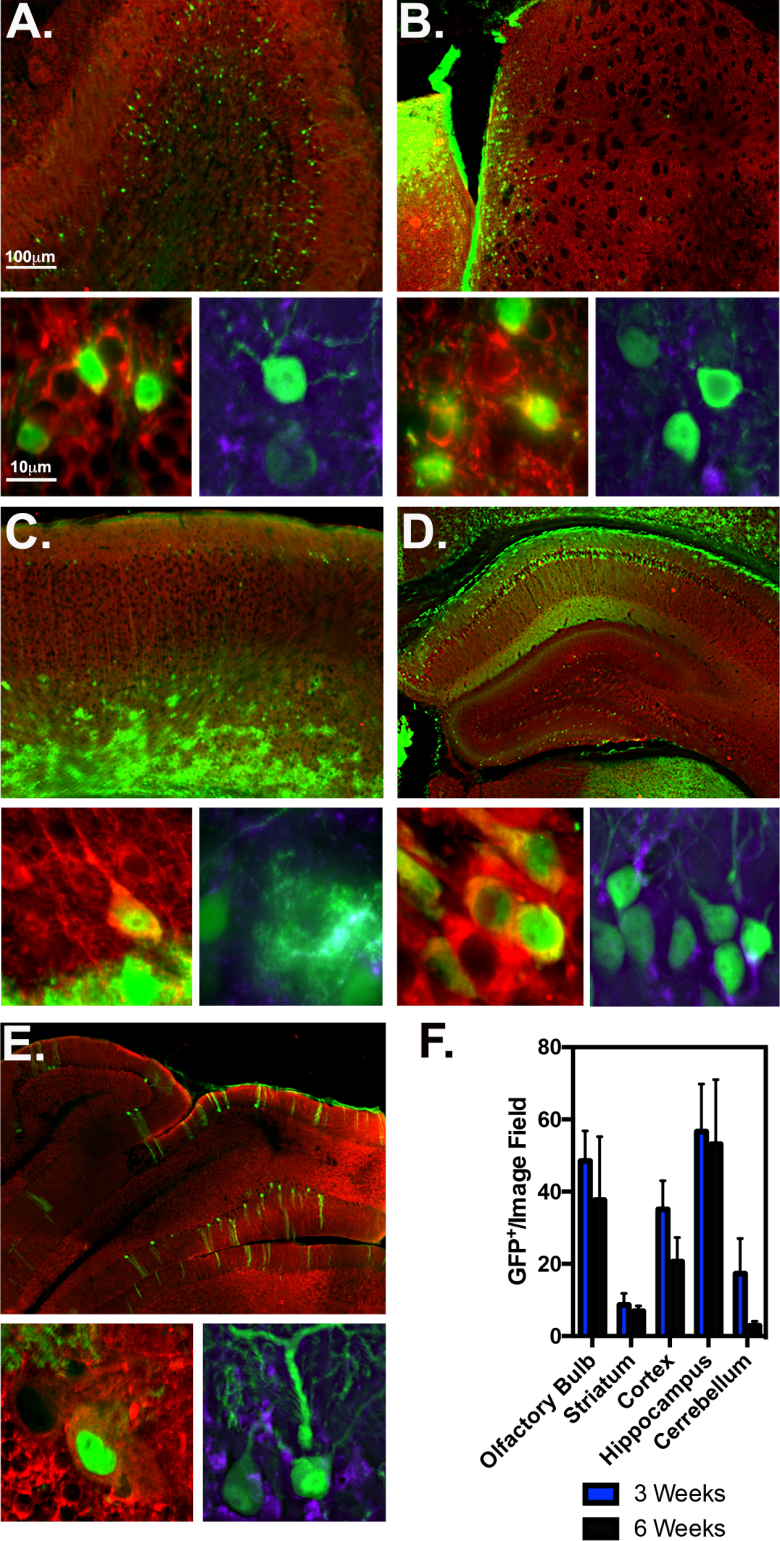
Tropism of AAV2/1 in multiple brain regions at 3 and 6 weeks post injection. AAV2/1 6-week injected tissue of multiple brain regions were immunostained for total neuronal marker MAP2 (red), astrocyte marker S100β (purple) and GFP (green) as depicted in representative 10X montage (top) and 100X high magnification images (bottom) of the olfactory bulb (**A**), striatum (**B**), motor cortex (**C**), hippocampus (**D**) and cerebellum (**E**). Notice; red/green co-localize to yellow, green/purple co-localize to cyan. (**F**) Each region was quantitated for GFP^+^/per 20X objective image field at 3 and 6-weeks post AAV2/1 injection (*n* = 3-4/serotype/time point).

To determine degree of AAV2/1-GFP tropism in neurons and astrocytes from multiple brain regions at 3 and 6 weeks post ICV injections, anatomical regions including the olfactory bulb, striatum, motor cortex, hippocampus and cerebellum were immunostained for anti-NeuN (red, neuronal marker) and S100β (purple, astrocytic marker) at 6 weeks post ICV (**Fig 3A–3E**). Mitral cells of the granular layer were primarily transduced, with no observed astrocytes in the olfactory bulb (**Fig 3A**). AAV2/1 had modest penetration from ventricular space to striatal neurons, without any GFP^+^ astrocytes observed in the caudate putamen (**Fig 3B**). Most GFP^+^ cells in the primary motor cortex were in Layer 6a-6b, and fewer GFP^+^ pyramidal neurons of cortical layers 5 and 2/3 (**Fig 3C**). Pyramidal layer neurons of CA1 hippocampus heavily expressed GFP, as well as some infected neurons in the molecular layer of the dentate gyrus (**Fig 3D**). Central lobules of the cerebellum had GFP^+^ Purkinje fiber neurons, no astrocytes were observed. Quantitative measurement of AAV2/1-GFP^+^ cells quantitated for each region/time point at 3 and 6 weeks are as follows: 47.5±11.7 and 37.7±17.5 in the olfactory bulb; 9.9+/-4.1 and 6.9+/-1.5 in the striatum; 38.2±10.3 and 20.7±6.7 in the cortex; 55.467±18.584 and 53.167±17.873 in the hippocampus; 21.8±12.3 and 2.9+/-1.6 in the cerebellum (*n =* 3/Time point) (**Fig 3F**).

### Quantitation of AAVDJ8-GFP tropism 3 and 6 weeks post injection

**Fig 4:**
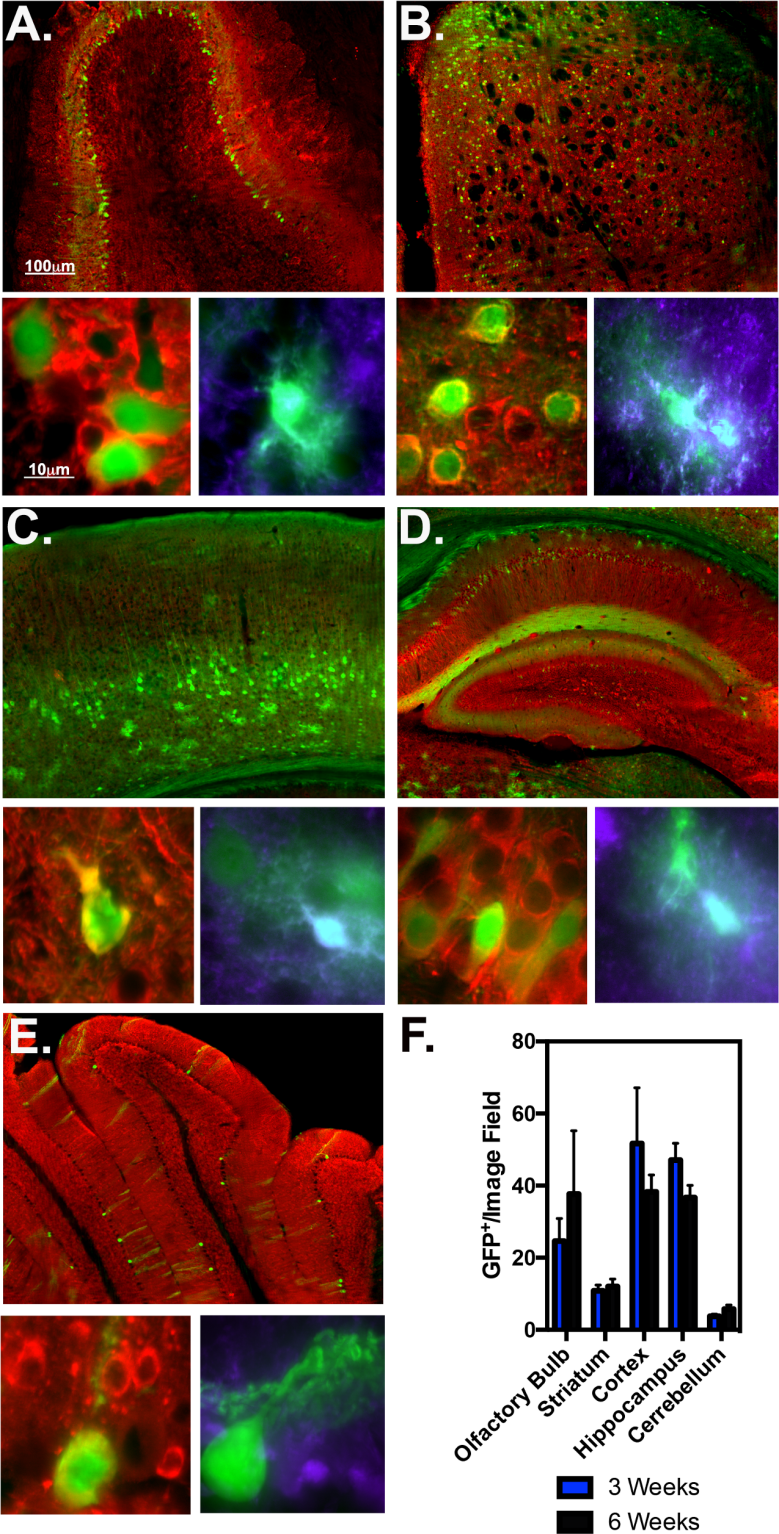
Tropism of AAVDJ8 in multiple brain regions at 3 and 6 weeks post injection. AAVDJ8 6-week injected tissue of multiple brain regions were immunostained for total neuronal marker MAP2 (red), astrocyte marker S100β (purple) and GFP (green) as depicted in representative 10X montage (top) and 100X high magnification images (bottom) of the olfactory bulb (**A**), striatum (**B**), motor cortex (**C**), hippocampus (**D**) and cerebellum (**E**). Notice; red/green co-localize to yellow, green/purple co-localize to cyan. (**F**) Each region was quantitated for GFP^+^/per 20X objective image field at 3 and 6-weeks post AAVDJ8 injection (*n* = 3/serotype/time point).

The tropism of AAVDJ8-GFP in multiple brain regions at 3 and 6 weeks post ICV injections was also monitored in the olfactory bulb, striatum, motor cortex, hippocampus and cerebellum (**Fig 4A–4E**). At 6 weeks post-ICV mitral layers of the olfactory bulb were heavily infected, including some observed AAVDJ8-GFP^+^ astrocytes (**Fig 4A**). Striatal neurons and astrocytes of the caudate putamen were primarily infected in the dorsal region of the striatum (**Fig 4B**). High AAVDJ8-GFP infection was observed in all layers of the primary motor cortex, with robust signal in pyramidal neurons of layer 5 and astrocytes of multiform layer 6 (**Fig 4C**). AAVDJ8-GFP^+^ pyramidal neurons of CA1 were observed in the pyramidal layer and infected astrocytes were observed in the dorsal hippocampal layer and stratum oriens (**Fig 4D**). Similar to AAV2/1, central lobules of the cerebellum GFP^+^ Purkinje fiber neurons were transduced but no GFP^+^ astrocytes were observed (**Fig 4E)**. AAVDJ8-GFP^+^ cells quantitated for each region/time point at 3 and 6 week are as follows: 24.7±6.3 and 29.0+/-2.8 in olfactory bulb; 10.9±1.6 and 12.0±2.1 in the striatum; 51.7±15.5 and 38.3+/-4.7 in the cortex; 47.1+/-4.6 and 36.7±3.4 in the hippocampus; 3.78±0.6 and 5.8±1.1 in the cerebellum (n = 3/Time point) (**Fig 4F**).

### Quantitation of AAV9-GFP tropism 3 and 6 weeks post injection

**Fig 5:**
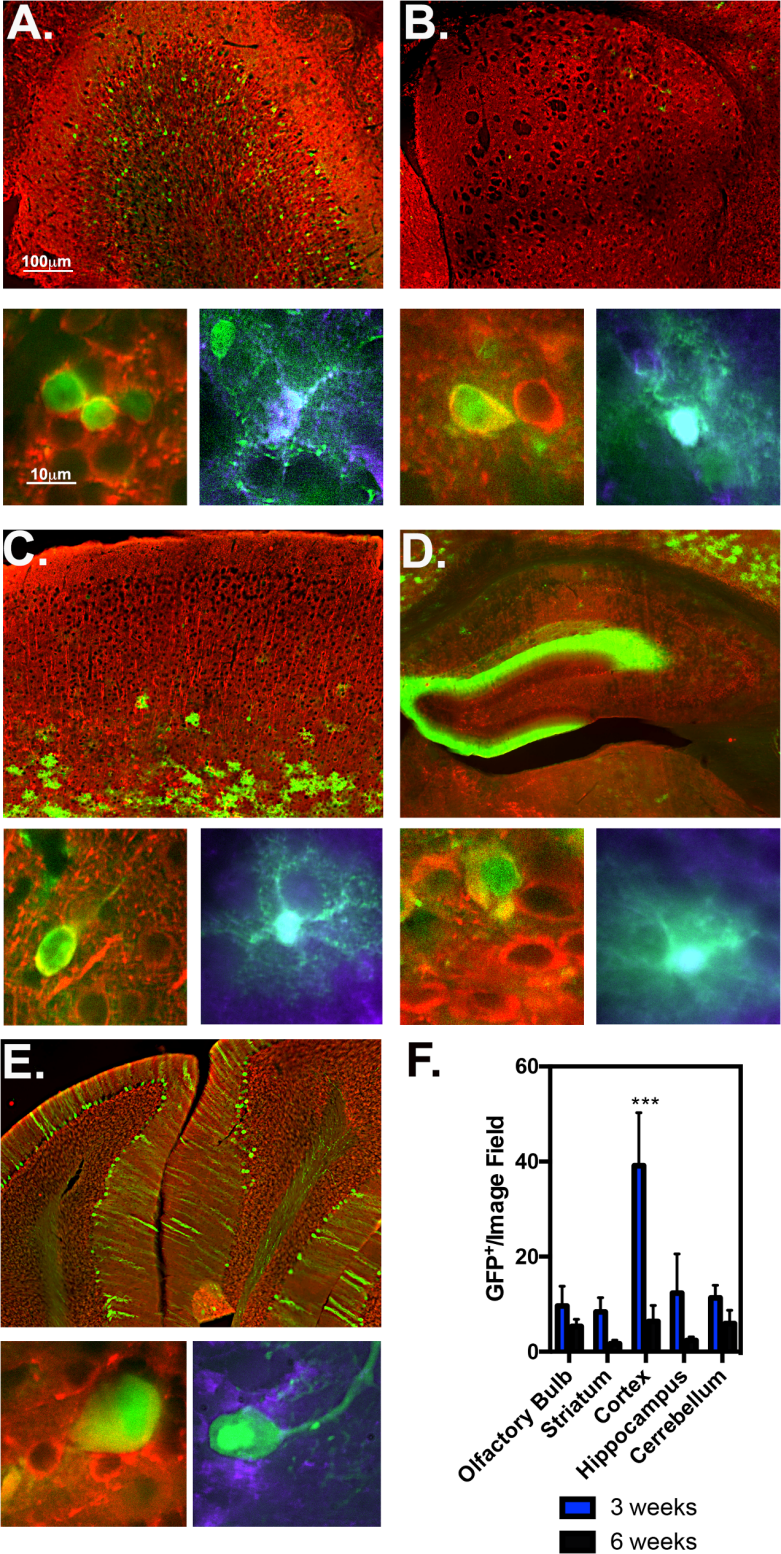
Tropism of AAV9 in multiple brain regions at 3 and 6 weeks post injection. AAV9 6-week injected tissue of multiple brain regions were immunostained for total neuronal marker MAP2 (red), astrocyte marker S100β (purple) and GFP (green) as depicted in representative 10X montage (top) and 100X high magnification images (bottom) of the olfactory bulb (**A**), striatum (**B**), motor cortex (**C**), hippocampus (**D**) and cerebellum (**E**). Notice; red/green co-localize to yellow, green/purple co-localize to cyan. (**F**) Each region was quantitated for GFP^+^/per 20X objective image field at 3 and 6-weeks post AAV9 injection (*n* = 3/serotype/time point; *p<0.05).

AAV9-GFP tropism was similarly monitored at 3 and 6 weeks post ICV injection. The olfactory bulb displayed comparable tropism to AAV2/1 with mainly infectivity in the mitral cells of the granular layer and no observed GFP^+^ astrocytes (**Fig 5A**). Minimum AAV9-GFP infection was present in the striatum, although infected neurons and astrocytes were identified within the caudate putamen region (**Fig 5B**). Both cell types were infected in the primary motor cortex, primarily neurons of layer 5 and astrocytes of layers 6a-b (**Fig 5C**). Neurons and astrocytes were minimally infected within the pyramidal layer of CA1 hippocampal region, and AAV9-GFP was noticeably expressed within the fiber tracts of the dentate gyrus (**Fig 5D**). Similar to AAV2/1 and AAVDJ8, Purkinje fiber neurons within the central lobules of the cerebellum were primarily infected, and no AAV9-GFP (+) astrocytes observed (**Fig 5E**). Transduction efficiencies of AAV9-GFP^+^ cells quantitated for each region/time point at 3 and 6 weeks are as follows: 9.6±4.2 and 5.3±1.5 in the olfactory bulb; 8.4±3.0 and 1.7±0.8 in the striatum; 39.1±11.1 and 6.4±3.3 in the cortex; 12.3±8.2 and 2.4±0.8 in the hippocampus; 11.3+/-2.7 and 5.9±2.8 in the cerebellum (***p< 0.001; *n* = 3/Time point) (**Fig 5F**).

### Analysis of AAV serotype-specific variation within the substantia nigra

**Fig 6:**
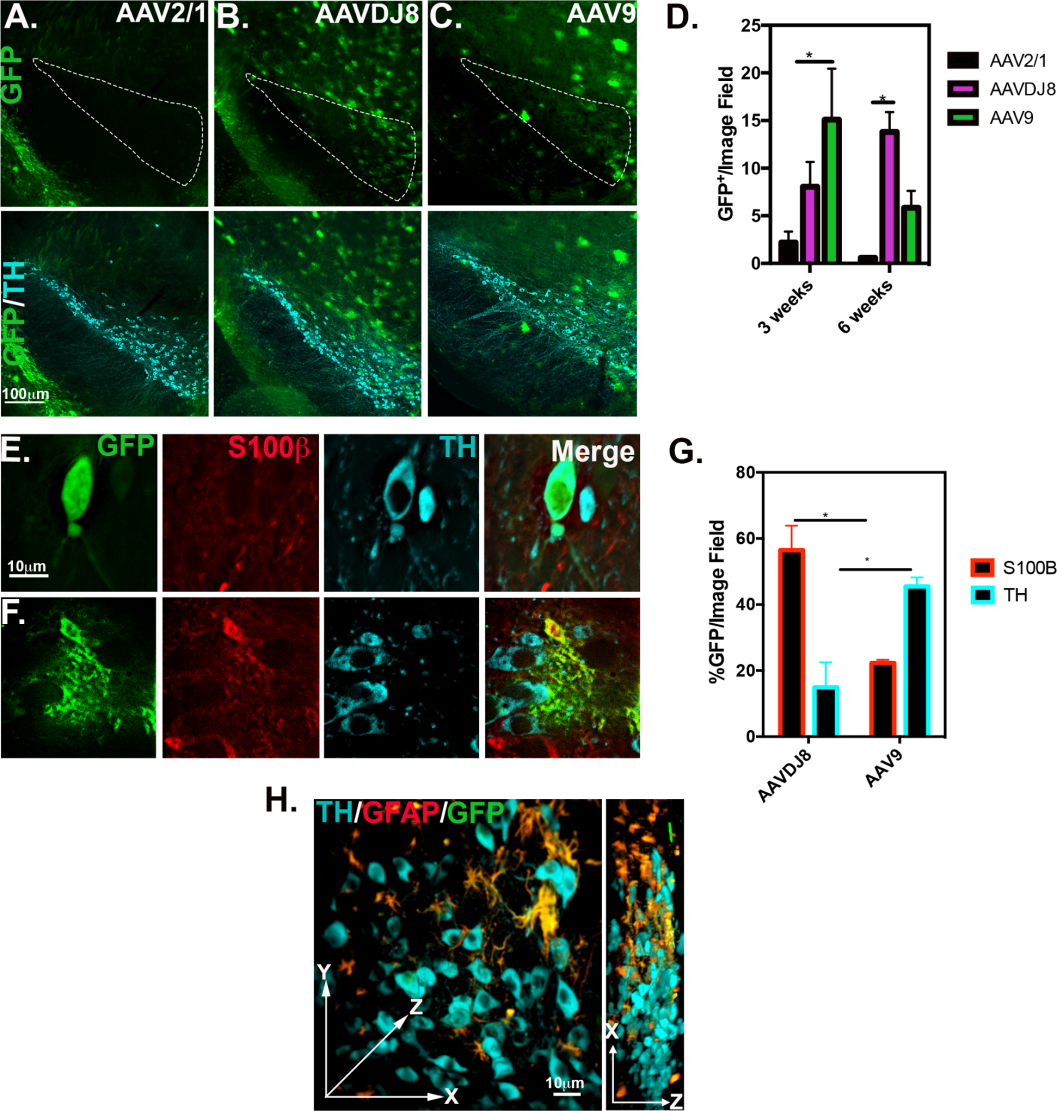
AAV9 and AAVDJ8 transduce DA neurons and astrocytes of the substantia nigra. 10X objective representative images of SN from 6-week tissue immunostained for TH (cyan) to visualize viral GFP expression (native) in the SN pars compacta (white outline) and SN pars reticulata for AAV2/1 (**A**), AAVDJ8 (**B**), and AAV9 (**C**) injected brains. (**D**) Total GFP^+^ cells/ 20X objective image field were quantitated for both 3 and 6-weeks post injection within the SN region (*n* = 3/serotype; *p<0.05). (**E**) AAV9 and (**F**) AAVDJ8 100X objective representative images immunostained for TH and S100β (red) within the SNpc. at 6-weeks post ICV. (**G**) % S100β-GFP^+^ and TH-GFP^+^ cells in the SNpc were quantitated/per 40X objective image field (*n* = 3/serotype; *p*<0.05). (**H**) Representative, three dimensional (3D) 40X objective montage image of clarified SN tissue from a AAVDJ8–GFP infected brain, immunostained for TH (cyan), GFAP (red) and GFP (green) in XYZ and XZ volumetric planes. Video of 3D projection can be found in **S1 Video**.

For further analysis/quantitation of the ventral midbrain region, specifically the substantia nigra (SN) was examined. Tissue sections were immunostained for dopamine neuron marker, tyrosine hydroxylase (TH), at 3 and 6 weeks post-ICV injection and quantitated for number of GFP^+^ cells. Representative IF images of AAV2/1, AAVDJ8 and AAV9 6 week SN tissue immunostained for dopamine neuron marker, tyrosine hydroxylase (TH) is depicted in **Fig 6A–6C**. Transduced GFP^+^ cells quantitated in the SNpc at 3 and 6 weeks are as follows: 2.2±14.3 and 0.5±0.16 for AAV2/1; 8.0± 2.6 and 13.8±2.1 for AAVDJ8; 15.1±5.3 and 5.8±1.8 for AAV9 (*p<0.05, *n* = 3/serotype/time point) (**Fig 6D**). Number of dopaminergic neurons transduced/per image field in the SN *pars compacta* (SNpc) at 6-weeks were measured by counting GFP^+^/TH^+^ co-localizing cells (**Fig 6E**). Transduction efficiency of astrocytes was also quantitated by number of GFP^+^/S100β^+^ co-localizing cells. AAVDJ8 transduction efficiency measured 56.4±7.5% S100β^+^/per image field cells and 14.9±7.6% and TH^+^ cells/per image field. AAV9 transduced 22.3±1.0% S100β^+^ cells/per field and 45.5±2.8% TH^+^ cells/per image field (*p<0.05, *n* = 3/serotype) (**Fig 6E–6G**). Pearson’s co-localization coefficient was also calculated for both cell markers as follows: 0.57±0.06 (GFP^+^/S100β^+^) and 0.46±0.04 (GFP^+^/TH^+^) for AAV9; 0.48±0.06 (GFP^+^/S100β^+^) and 0.54±0.05 (GFP^+^/TH^+^) for AAVDJ8 (25–42 GFP^+^ cells/over n = 3 animals/serotype; **Fig B in S1 File)**. Mean intensity of GFP fluorescence was also measured for both cell types as accordingly: 295.1±75.5 AU/per S100B^+^ cells and 309.6±65.8 AU/per TH^+^ cells for AAV9; 1207.0±142.2 AU/per S100B^+^ cells and 1157.0±105.9 AU/per TH^+^ cells for AAVDJ8 (25–42 GFP^+^ cells/over *n* = 3 animals/serotype; *p*<0.0001****; **Fig B in S1 File**). CLARITY tissue transmutation was performed on SN tissue to visualize AAVDJ8 transduction in 3D volumetric space. Clarified tissue was co-immunostained for TH (cyan), GFAP (red) and GFP (green), with expression of GFP primarily confined to astrocytes, noted by co-localization of red and green fluorescence (yellow) (**Fig 6H, S1 Video**).

### AAVDJ8-GFAP-mCherry specifically targets astrocytes of SNpc

**Fig 7:**
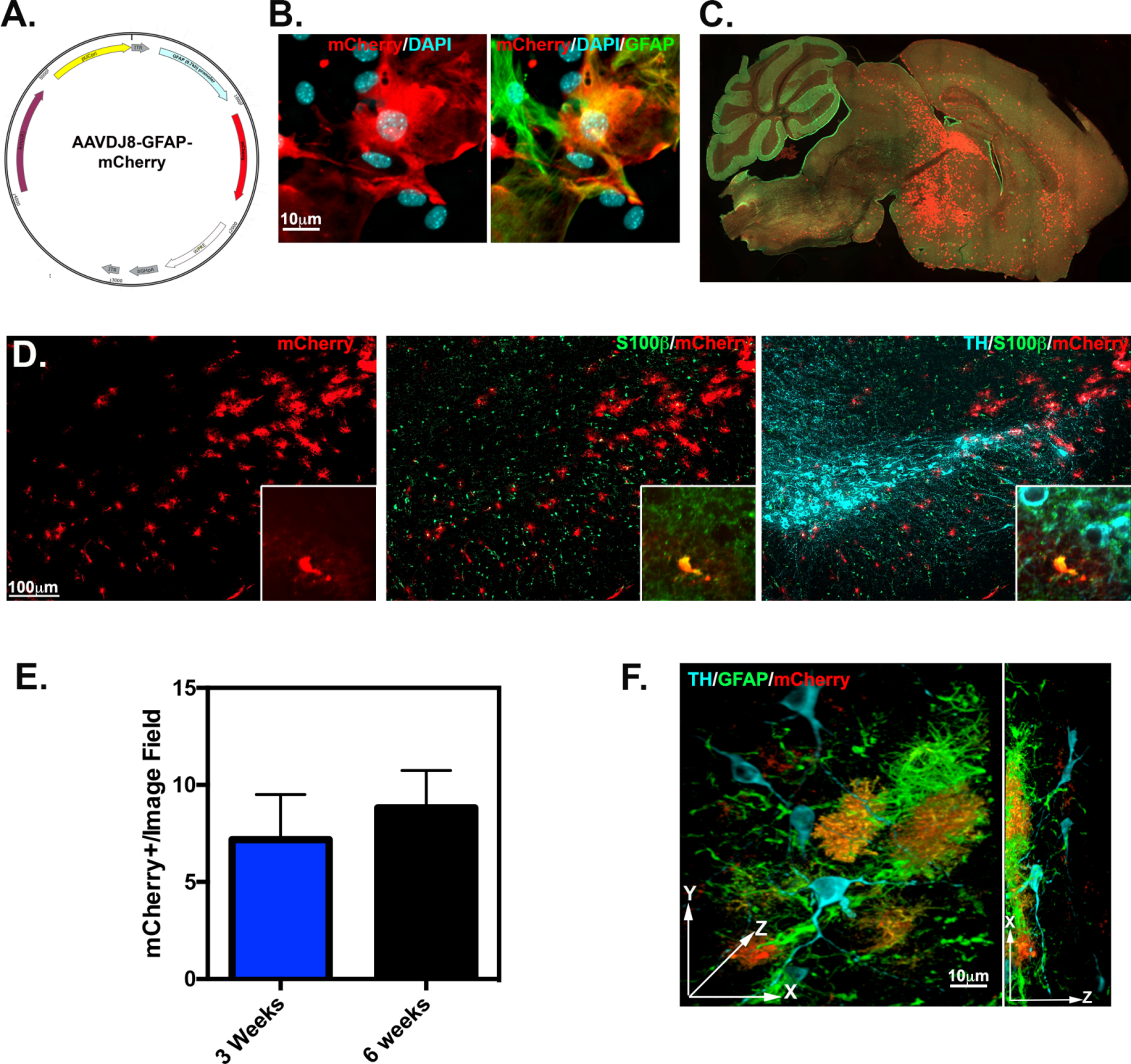
AAVDJ8-GFAP-mCherry targets exclusively astrocytes in the substantia nigra. (**A**) Astrocyte-specific promoter, GFAP, was incorporated into suitable serotype AAVDJ8 to drive expression of fluorescent reporter, mCherry for targeting astrocytes, as depicted in plasmid AAV vector map. (**B**) Primary astrocyte cultures were transduced with AAVDJ8-GFAP-mCherry to confirm astrocyte transduction efficiency *in vitro* as depicted in representative 100X objective images of immunostaining for mCherry(red) and GFAP(green), 83.2%±6.5 GFAP^+^/mCherry^+^ quantitated. (**C**) mCherry (red) and S100β(green) immunofluorescence is visualized in 10X objective montage image of sagittal cross section from AAVDJ8-GFAP-mCherry 3-week infected brain. (**D**) AAVDJ8-GFAP-mCherry expression in 10X objective image of SN region immunostained for mCherry (red), S100β(green), and TH (cyan) from 3 week infected brain, 100X objective inset images represent colocalization of S100β^+^/mCherry^+^ cells. (**E**) AAVDJ8-GFAP-mCherry expression levels were quantitated for mCherry^+^cells/20X objective image field at 3 and 6 weeks post ICV (*n* = 3/serotype, groups were not significant). (**F**) 3D volumetric view in XYZ and XY planes for CLARITY immunofluorescent image stained for TH (cyan), GFAP (green) and mCherry (red) within the SNpc. AAVDJ8-GFAP-mCherry infected astrocytes are exclusiveling co-localizing with GFAP^+^ cells within the vicinity of dopaminergic neurons. Video of 3D projection can be found in **S2 Video**.

After determining the suitability of AAVDJ8 to transduce astrocytes within the SNpc, we used this serotype to express red fluorescent mCherry under the control of a truncated version of the astrocyte-specific *gfap* promoter (0.7kb) that is active and can be efficiently cloned into AAV (**Fig 7A**). Strong expression of AAVDJ8-GFAP-mCherry was observed in primary cortical astrocyte cultures at 83.2%±6.5 mCherry^+^/GFAP^+^ as depicted in representative images in **Fig 7B**. AAVDJ8-GFAP-mCherry was also tested *in vivo* by ICV to observe cell-specificity and tropism throughout the brain. By 3 weeks post-injection, high levels of mCherry expression were detected within cortical, hippocampal, thalamic and midbrain areas, as depicted in whole brain images of sagittal cross sections (**Fig 7C**). To determine if there was similar penetrance to the SN as AAVDJ8-eGFP, we imaged immunostained SN tissue for TH, S100β and mCherry, shown in the representative images in **Fig 7D**. Images of tissue at 3 weeks post-injection were quantitated for the number of transduced astrocytes within the SN, indicating 80.3± 6.3 mCherry^+^/S100β^+^ cells/image field. No TH^+^ neurons were found to express mCherry. Mice injected with AAVDJ8-GFAP-mCherry were also aged for 6-weeks to determine the stability of expression with this vector. No significant difference was observed between time points; mCherry^+^cells/image field was quantitated at 7.2±2.3 and 8.8±1.9 at 3 and 6 weeks respectively (*n* = 3/time point; **Fig 7E**). GFAP^+^-mCherry^+^ co-localization within the vicinity of dopaminergic neurons is visualized similar to AAVDJ8-GFP by a 3D volumetric view of the clarified SNpc tissue at ~4.5 times thicker (180μm) than typical tissue sections cut for cell quantitation (**Fig 7F**; **S2 Video)**.

## Discussion

AAV vectors are valuable tools for transduction of both mitotic and post-mitotic cells of the CNS. The high levels of tropism in multiple brain regions permit expression of a diverse array of transgenes suitable for mechanistic investigation of basic biological function as well as neurological disease. AAV serotype transduction differences have been highly studied for primary cell culture and for gene expression *in vivo*. However, there are few studies that provide a systematic approach for selection of AAV serotypes to achieve both *in vitro* and *in vivo* gene expression in murine neurons and astrocytes in a region-specific fashion. By use of multiple different AAV-GFP serotypes with CMV/CAG promoters, we demonstrate differences in infectivity and gene expression in primary astrocytes and neurons, as well as tropism in multiple regions of the brain, stability of AAV-GFP expression between 3–6 weeks post ICV injection and expression of AAV-GFP in ventral midbrain areas. In addition, we identified an AAV serotype to transduce astrocytes within the SNpc through evaluation of AAVDJ8-GFAP-mCherry.

Serotype transduction differences have been previously reported in rat primary cortical neuronal cultures utilizing naturally occurring serotypes and engineered capsids. Howard et al. found AAV1-CMV-GFP to have the highest expression of GFP compared to other naturally occurring serotypes in neuronal cultures [6]. Accordingly, we screened both the naturally occurring AAV1-CMV-GFP serotype in comparison to engineered hybrid serotypes AAV2/1-CAG-eGFP, AAVDJ-CAG-eGFP, AAV8-CMV-GFP, AAVDJ8-CAG-eGFP, AAV9-CMV-GFP and AAVDJ9-CAG-eGFP to determine transduction efficiency in both MAP2^+^ and GFAP^+^ cells in culture. AAV2/1 transduced the most MAP2^+^ neurons and GFAP^+^ astrocytes in culture based on immunostaining. Additionally, according to daily measurements of GFP in cultured neurons, AAV2/1 was the most rapid and highest expressing serotype by DIV 7. Hence, AAV2/1 was the most efficient for transducing cells *in vitro* amongst the serotypes tested (**Fig 1**). AAV2/1 has been utilized to target neurons for anti-inflammatory effects of dominant-negative chemokine CCL2 mutant, interleukin-10 (IL-10) in mouse models of Alzheimer’s Disease (AD) and brain-derived neurotrophic factor (BDNF) in rat models of Huntington’s Disease [22] [23] [24]. High transduction efficiency has been reported in regions of the basal ganglia in rat and non-human primate animal models by stereotaxic injection using AAV2/1 [25] [26,27]. In comparison to AAV1 sagittal cross-sections, the patterns of transgene expression were drastically different with ICV delivery. AAV1 transduction was limited to the ependymal cells of the choroid plexus, whereas AAV2/1-GFP expression was observed throughout the brain (**Figs B and D in S1 File**). Restriction of AAV1 to the ventricular epithelia when delivered via ICV is consistent with studies by JY Kim et al. 2013 and further supports our findings [14]. These data suggest the ITR *cis*-acting elements of AAV2 and CAG promoter within pseudotype AAV2/1 are crucial for transduction efficiency in culture and penetration from ventricle to parenchymal space. With these findings, we selected AAV2/1 for further quantitative analysis in multiple brain regions.

AAVDJ8 displayed promising results in glial cultures, comparable to AAV2/1 (**Fig 1O**). Wide distribution and higher levels of GFP expression were noticeable throughout the brain compared to AAV8 (**Fig 2D, Fig D in S1 File**). It was previously demonstrated that the AAVDJ8 pseudo-serotype produces ~10-fold higher titers than AAV2/1 and more efficiently drives expression of red fluorescent protein (RFP) in neurons of the rat amygdala [28]. Prior studies have also indicated that AAV2/8 is an effective vector for targeting neurons of the nigrostriatal system in neurodegenerative studies when administered via stereotaxic injection directly to the substantia nigra in rats [25,29]. Also, AAV8-based vectors have shown selective expression in astrocytes of the spinal cord, hippocampus, striatum and substantia nigra of adult rats when used with human GFAP promoter to drive transgene expression [30,31]. Our findings with AAVDJ8-CAG-eGFP are consistent with these studies and support the use of the AAVDJ8 serotype to target neurons and astrocytes of specific brain regions. Furthermore, AAVDJ8 was a suitable serotype for GFAP-mCherry design to restrict transgene expression in astrocytes.

AAV9 was chosen for further investigation based initially on the ability to transduce neurons *in vitro* and from reports that it can target multiple cell types *in vivo*[12,15,32,33]. AAV9 and AAVDJ9 had the same efficiency in primary neuronal cultures but AAV9 transduced 5% more astrocyte than AAVDJ9 in culture (**Fig 1**). AAVDJ9 did depict a high level and wide distribution of transgene expression throughout most brain regions evaluated, yet had lower levels of penetrance to the ventral midbrain regions when compared to AAV9 (**Figs D and E in S1 File)**. In previous studies, AAV9 has been used to target hippocampal neurons when delivered via stereotaxic injection in mice[12]. However, it was also reported that AAV9 crosses the blood brain barrier when delivered intravascularly and targets neurons in neonatal mice but then expresses in astrocytes in adult mice [32]. In contrast, Gray et al. asserted that AAV9 preferentially targets neurons in adult mice when utilizing the same delivery method and promoter/GFP reporter[33]. Interestingly, AAV9 has been used to target TH^+^ neurons of the SNpc under control of the human synapsin (hSYN1) promoter to drive GFP expression after neonatal delivery via ICV[15]. Based on these findings and our data, we selected AAV9 for further quantitative analysis in multiple brain regions.

Since initial ICV studies, this method has been tested and optimized for timing of injection and serotype comparisons[14,18]. It was found that when injecting at different time points (0-72hrs), AAV2/1 had broadest distribution throughout the brain at P0, whereas AAV2/8 and AAV2/9 transduced independently of the age at which they were injected [5]. For the most efficient viral dissemination and consistency, we administered all AAV serotypes at P0. At 3-weeks post injection, AAV2/1 transduced the most cells of the olfactory bulb, hippocampus and cerebellum (**Table 1)**. AAVDJ8-GFP^+^ cells were most prevalent in the striatum and cortex; and AAV9 transduced the most cells of the substantia nigra (**Figs 3F–5F, Table 1**). Based on previous studies, ICV-delivered AAVs have been proven to be stable for up to 1-year post injection[12,34]. Interestingly, we noticed AAV-GFP expression decreased in many of the regions examined infected at 6-week post injection. AAV9-GFP and AAV2/1-GFP lost expression in all brain regions at 6-weeks post-ICV, whereas AAVDJ8-GFP expression decreased only in the cortex and hippocampus. The reduction in AAV transgene expression is consistent with other studies utilizing CMV-promoter based AAVs[11,35]. Explanations for the observed loss of AAV transgene expression over time could be attributed to cell turnover within the given regions or that CMV promoters are prone to transcriptional inactivation by DNA methylation during viral latency [10]. It was also reported that AAV transgene expression and persistence is improved when utilizing an endogenously expressed promoter-neuron-specific enolase (NSE)-containing construct compared to a CMV-containing construct [11].

AAV2/1 had very minimal penetrance to the ventral midbrain area at both time points. AAVDJ8 was persistent in expression and AAV9 decreased from ~15 to ~6 GFP^+^/image field in the SNpc. In support of our findings, previous studies reported ~46% of neurons in the SN were transduced with AAV9-hSyn-GFP at 6 weeks post ICV[15]. Correspondingly, we quantitated ~45% TH^+^ neurons and ~22% S100β^+^ astrocytes/per field with AAV9 (**Fig 6D and 6H**). Also, AAV9 transduced more TH^+^ neurons than AAVDJ8, whereas AAVDJ8 transduced more astrocytes (**Fig 6G**). Co-localization (GFP +TH or S100B) and intensity values of GFP signal were also measured. As expected, AAVDJ8 had significantly higher GFP signal in both cell types due to the construct containing CAG promoter and eGFP, but Pearson’s colocalization coefficients were not significantly different between serotypes, indicating higher GFP signal did not interfere with quantitative analysis (**Fig B in S1 File**). Based on morphological co-localization studies, we noted a lack of co-localization of GFP with IBA1^+^ cells using any serotype (see **Fig F in S1 File)**This is consistent with other reports using these AAV vectors, which identified oligodendrocytes as an additional cell type transduced by these vectors rather than microglia [5,12,31]. Although the levels of AAV infectivity in the SNpc by ICV delivery were not as robust compared to the alternative SN stereotaxic injection method, there was evident penetration with AAV9 and AAVDJ8 within this region of the brain that suggests these two serotypes would be suitable for targeting specific cells of the SNpc via ICV administration (**Fig 6H**).

Several attempts to treat PD patients with AAVs have been made. Safety/feasibility in clinical phases has shown potential gene therapy with AAV2- Glial Derived Neurotrophic Factor (GDNF/neurturin) [36,37]. However, results from a phase 2 randomized trial declared there was no significant therapeutic benefit and 3 patients developed tumors [38,39]. Another failed attempt tested AAV2- aromatic _L_-amino acid decarboxylase (AADC) in PD patients, but suggested a new trial was needed to confirm efficacy [40]. Current clinical trials have predominately only used neurotropic serotype AAV2, which do not target astrocytes. Drinkut et al., confirmed injection of AAV5-hGFAP(2.2 kb) for astrocyte-specific GDNF overexpression in the mouse provided the same neuroprotective efficacy as neuron-derived GDNF, and suggests more AAV based therapies should be targeted towards astrocytes [41]. Additionally, neuroinflammatory mechanisms that cause dopaminergic neuronal loss during PD progression are heavily mediated by astrocyte activation [42]. Here, we report AAVDJ8 to be a stable serotype to transduce astrocytes of the SNpc by ICV at 6 weeks (**Fig 6G and 6H**). By use of enhanced CLARITY microscopy, AAVDJ8-eGFP expression can be visualized in brain tissue at ~2 times greater z-dimensions primarily in GFAP^+^ astrocytes in a 3D-volumetric view of the SN (**Fig 6E, S1 Video**).

To further limit AAV transgene to astrocytes, we tested the use of truncated 681 bp human-*gfap* (gfaABC1D) promoter in AAVDJ8-GFAP-mCherry constructs by ICV delivery (**Fig 7A**). The gfaABC1D promoter was previously designed to have two-fold greater activity than full-length (2.2kb) promoter and with much smaller size, ideal for the limited cloning capacity in AAV vectors[43]. The full-length human-*gfap* was previously validated to transduce astrocytes with AAV2/1 striatal neonatal injections and selectivity of the 1.74 kb GFAP promoter has been tested with combined serotype AAV2/5/7/8/9 by cortical injection. More specifically, gfaABC1D promoter has been used with AAV9 via intramuscular delivery [44–46]. The data presented here are the first report that AAVDJ8-GFAP-mCherry efficiently and selectively expresses in cultured astrocytes and in the SNpc following neonatal ICV delivery in mice (**Fig 7B and 7D**). In culture, longer DIV incubation time and higher titer was required for robust mCherry expression, most likely because the GFAP promoter is weaker than CAG/CMV in culture. However, *in vivo* we noticed similar distribution patterns of AAVDJ8-GFAP-mCherry compared to AAVDJ8-eGFP in sagittal plane views (**Fig 7C, Fig 2G**). There was also comparable number of mCherry^+^ cells/image field at 3–6 weeks to GFP^+^ cells/image field in the SN. AAVDJ8-eGFP had slightly higher number of transduced cells in this region at 6 weeks due to observed neurons transduced with this vector and no identified TH^+^/mCherry^+^ cells with AAVDJ8-GFAP-mCherry **(Fig 6D–6G, Fig 7D–7F**). To fully visualize exclusive astrocyte-specific targeting of AAVDJ8-GFAP-mCherry we also conducted CLARITY immunofluorescent imaging, this time at ~4.5 times greater z-stack thickness than normal tissue samples for a better view of AAV penetrance within the SN (**Fig 7F, S2 Video**).

Serotype differences *in vivo* were partially attributed to the ability to penetrate into the parenchymal space by ICV administration method, however there were differences in brain regions and cell specific transduction patterns compared to *in vitro* results. For example, AAV2/1 had the highest transduction efficiency *in vitro*, but did not virally express as well compared to AAV9/AAVDJ8 in the SNpc or was as stable in other regions compared to AAVDJ8 at 6-weeks. The observed differences of serotype transduction efficacies *in vitro* compared to *in vivo* are a common issue when determining ideal serotypes for specific brain regions and further supports the rationale to proceed past an initial *in vitro* assessment if the viral models are intended to be used *in vivo*[4,47]. Lastly, with selection of the appropriate combination of serotype and promoter, targeting specific cell types within the SNpc can be accomplished for transgene expression for studies of biological mechanism or disease intervention.

## Supporting information

S1 File Figures A-F are included in uploaded supporting information. **Fig A ICV Mock injected brain**. Mock ICV injected brains were rapidly dissected and imaged for bright field on stereo dissection microscope to confirm ICV technique targeted the (**A**) lateral ventricles (LV) and spread to (**B**) the mesencephalic aqueduct (AQ). **Fig B Quantitation of astrocyte/neuron co-localization of AAV9 and AAVDJ8 in the SNpc** (**A**) Pearson’s correlation coefficients were measured for S100β^+^/GFP^+^ and TH^+^/GFP^+^ co-localizing cells identified in the SNpc from 40x-objective images of AAV9-GFP and AAVDJ8-eGFP 6-week infected brain tissue. AAV-GFP intensity values were also measured in (**B**) TH^+^ and (**C**) S100β^+^ cells (25–42 GFP^+^ cells/over *n* = 3 animals/serotype; *p*<0.0001****). **Fig C Intrinsic GFP detection of whole brain AAV infected brains** Lateral views of GFP fluorescence in grayscale compared to uninjected control of (**A**) AAV2/1-eGFP, (**B**) AAVDJ8-eGFP, and (**C**) AAV9-GFP 3-week infected whole brains (images are representation of *n* = 3/serotype). **Fig D Sagittal plane views of other AAV serotypes tested by ICV** All serotypes screened *in vitro* were also tested *in vivo* by ICV administration. (**A**) Uninjected control (**B**) AAVDJ-CAG-eGFP, (**C**) AAV1-CMV-GFP, (**D**) AAV5-CMV-GFP, (**E**) AAV8-CMV- GFP, and (**F**) AAVDJ9-CAG-eGFP at 3-weeks post injection were observed for native GFP fluorescence as depicted in representative 10x-objective montage images of sagittal cross sections (Images are representation of *n* = 3/serotype). **Fig E AAV9 has better penetrance to the SN than AAVDJ9 by ICV** Immunofluorescence for TH (red) and intrinsic GFP (green) are depicted in 10X objective images of the SN from (**A**) AAV9 and (**B**) AAVDJ9 3-week infected brains (Representation of *n* = 3/serotype). **Fig F AAVDJ8-GFP and AAVDJ8-GFAP-mCherry do not transduce microglia in the SNpc** 64X objective immunofluorescent images of (**A**) anti-mCherry (red) and anti-IBA1 (green) depict no co-localization of AAVDJ8-GFAP-mCherry infected cells. (**B**) Anti-GFP (green) and anti-IBA1(red) immunoflourescent images also depict no co-localization of AAVDJ8-GFP (Images are representation of *n* = 3/serotype).
(PDF)

S1 Video 3D volumetric view of AAVDJ8-CAG-eGFP infected SN. (AVI)

S2 Video 3D volumetric view of AAVDJ8-GFAP-mCherry infected SN. (AVI)
