# Supplementary material for: Cellular selectivity of AAV serotypes for gene delivery in neurons and astrocytes by neonatal intracerebroventricular injection
Source: PLoS One. 2017 Dec 15;12(12):e0188830. doi: 10.1371/journal.pone.0188830 (PMC5731760; doi:10.1371/journal.pone.0188830)
Supplement: S1 File — Figures A-F are included in uploaded supporting information. Fig A ICV Mock injected brain. Mock ICV injected brains were rapidly dissected and imaged for bright field on stereo dissection microscope to confirm ICV technique targeted the (A) lateral ventricles (LV) and spread to (B) the mesencephalic aqueduct (AQ). Fig B Quantitation of astrocyte/neuron co-localization of AAV9 and AAVDJ8 in the SNpc (A) Pearson’s correlation coefficients were measured for S100β+/GFP+ and TH+/GFP+ co-localizing cells identified in the SNpc from 40x-objective images of AAV9-GFP and AAVDJ8-eGFP 6-week infected brain tissue. AAV-GFP intensity values were also measured in (B) TH+ and (C) S100β+ cells (25–42 GFP+ cells/over n = 3 animals/serotype; p<0.0001****). Fig C Intrinsic GFP detection of whole brain AAV infected brains Lateral views of GFP fluorescence in grayscale compared to uninjected control of (A) AAV2/1-eGFP, (B) AAVDJ8-eGFP, and (C) AAV9-GFP 3-week infected whole brains (images are representation of n = 3/serotype). Fig D Sagittal plane views of other AAV serotypes tested by ICV All serotypes screened in vitro were also tested in vivo by ICV administration. (A) Uninjected control (B) AAVDJ-CAG-eGFP, (C) AAV1-CMV-GFP, (D) AAV5-CMV-GFP, (E) AAV8-CMV- GFP, and (F) AAVDJ9-CAG-eGFP at 3-weeks post injection were observed for native GFP fluorescence as depicted in representative 10x-objective montage images of sagittal cross sections (Images are representation of n = 3/serotype). Fig E AAV9 has better penetrance to the SN than AAVDJ9 by ICV Immunofluorescence for TH (red) and intrinsic GFP (green) are depicted in 10X objective images of the SN from (A) AAV9 and (B) AAVDJ9 3-week infected brains (Representation of n = 3/serotype). Fig F AAVDJ8-GFP and AAVDJ8-GFAP-mCherry do not transduce microglia in the SNpc 64X objective immunofluorescent images of (A) anti-mCherry (red) and anti-IBA1 (green) depict no co-localization of AAVDJ8-GFAP-mCherry infected cells. (B) Anti-GFP (green) an [file pone.0188830.s001.pdf]

S1 File- Supplemental Figures

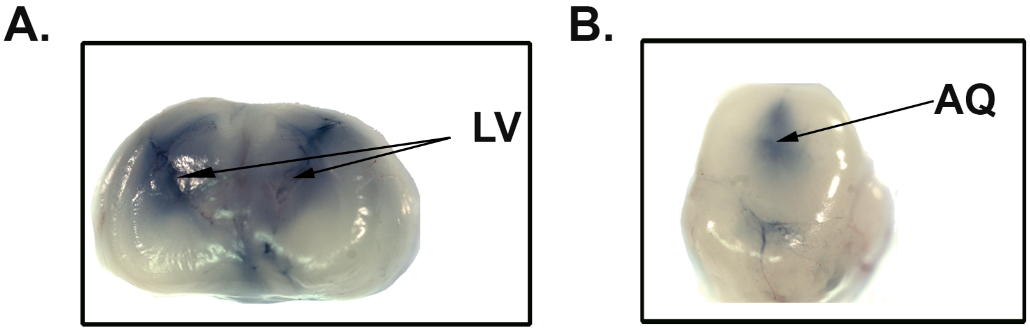

Fig A. ICV Mock injected brain. Mock ICV injected brains were rapidly dissected and imaged for bright field on stereo dissection microscope to confirm ICV technique targeted the (**A**) lateral ventricles (LV) and spread to (**B**) the mesencephalic aqueduct (AQ).

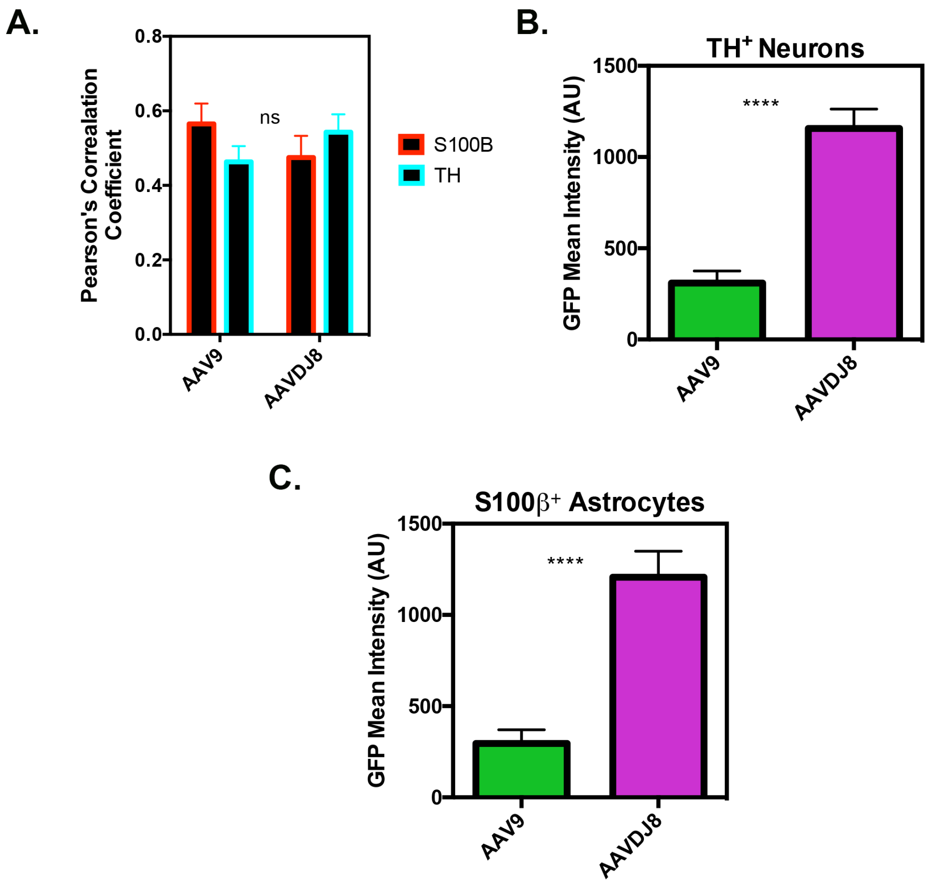

Fig B. Quantitation of astrocyte/neuron co-localization of AAV9 and AAVDJ8 in the SNpc (**A**) Pearson's correlation coefficients were measured for S100β$^+$/GFP$^+$ and TH$^+$/GFP$^+$ co-localizing cells identified in the SNpc from 40x-objective images of AAV9-GFP and AAVDJ8-eGFP 6-week infected brain tissue. AAV-GFP intensity values were also measured in (**B**) TH$^+$ and (**C**) S100β$^+$ cells (25-42 GFP$^+$ cells/over n=3 animals/serotype; p<0.0001****).

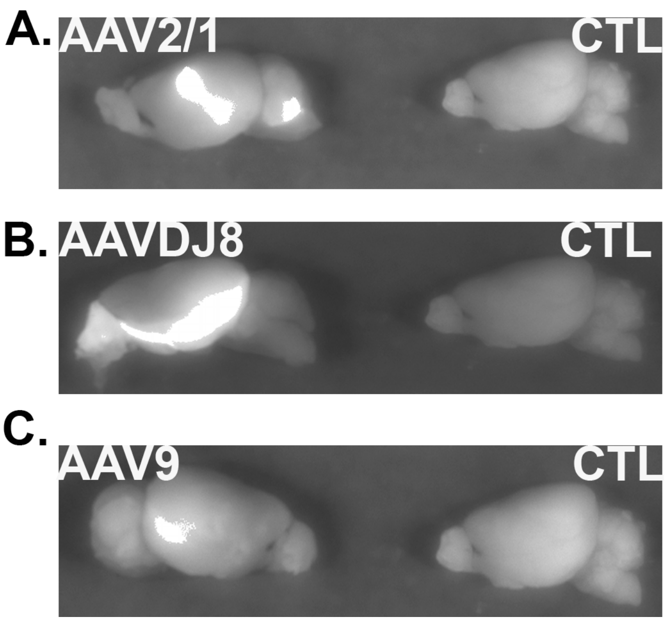

Fig C. Intrinsic GFP detection of whole brain AAV infected brains

Lateral views of GFP fluorescence in grayscale compared to uninjected control of (**A**) AAV2/1-eGFP, (**B**) AAVDJ8-eGFP, and (**C**) AAV9-GFP 3-week infected whole brains (images are representation of *n*=3/serotype).

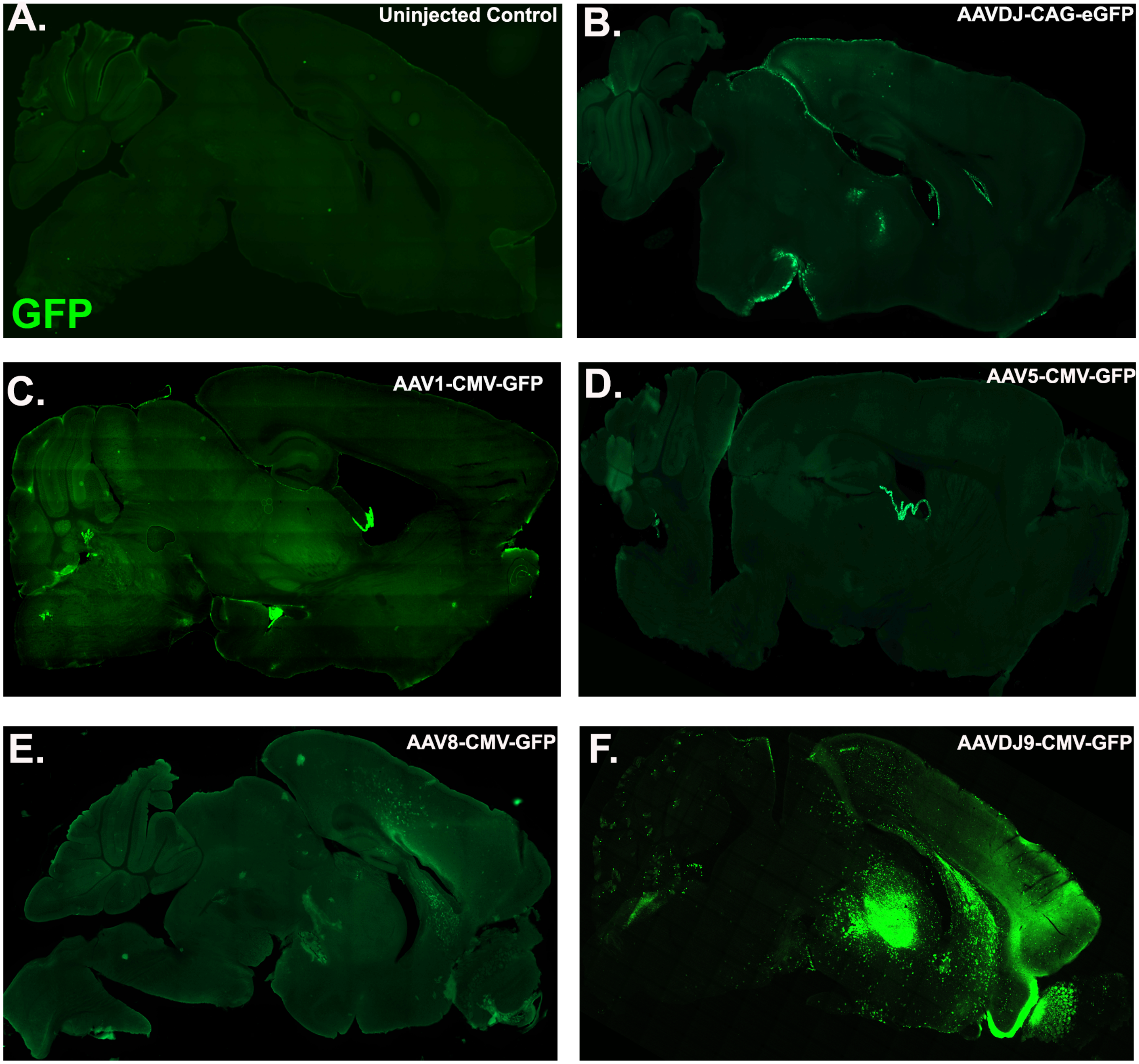

Fig D. Sagittal plane views of other AAV serotypes tested by ICV

All serotypes screened *in vitro* were also tested *in vivo* by ICV administration. (**A**) Uninjected control (**B**) AAVDJ-CAG-eGFP, (**C**) AAV1-CMV-GFP, (**D**) AAV5-CMV-GFP, (**E**) AAV8-CMV- GFP, and (**F**) AAVDJ9-CAG-eGFP at 3-weeks post injection were observed for native GFP fluorescence as depicted in representative 10x-objective montage images of sagittal cross sections (Images are representation of *n*=3/serotype).

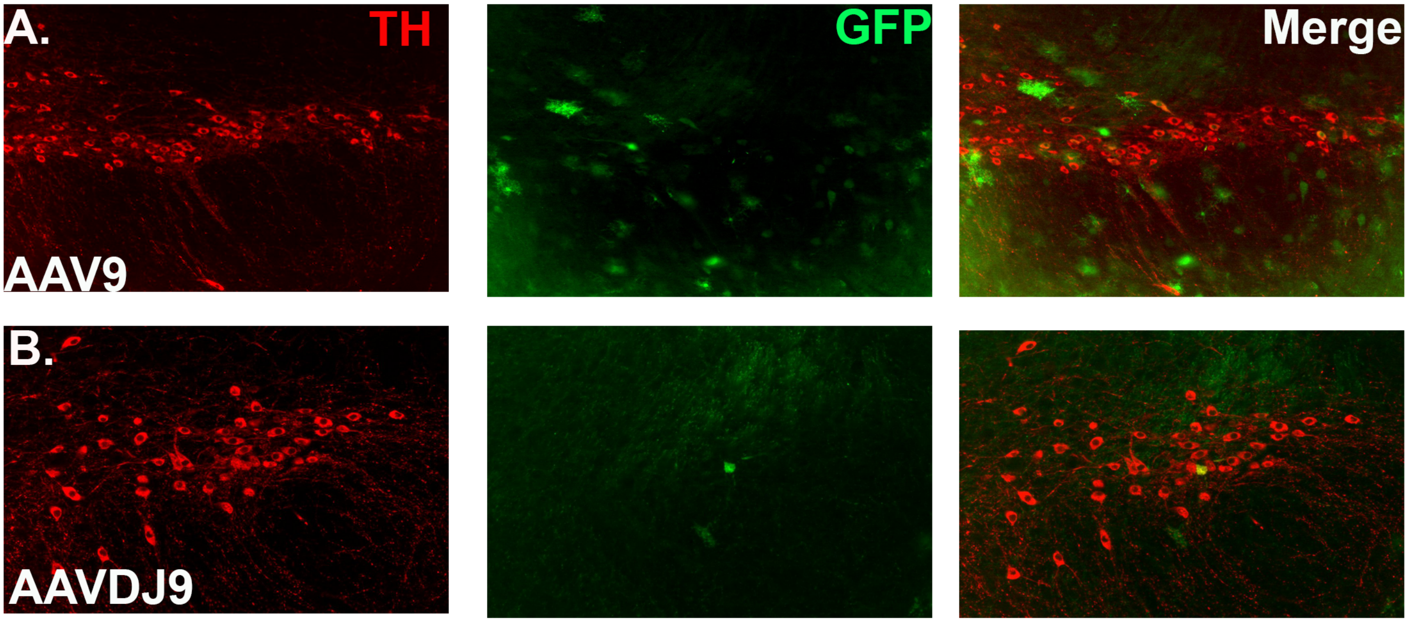

Fig E. AAV9 has better penetrance to the SN than AAVDJ9 by ICV

Immunofluorescence for TH (red) and intrinsic GFP (green) are depicted in 10X objective images of the SN from (**A**) AAV9 and (**B**) AAVDJ9 3-week infected brains (Representation of *n*=3/serotype).

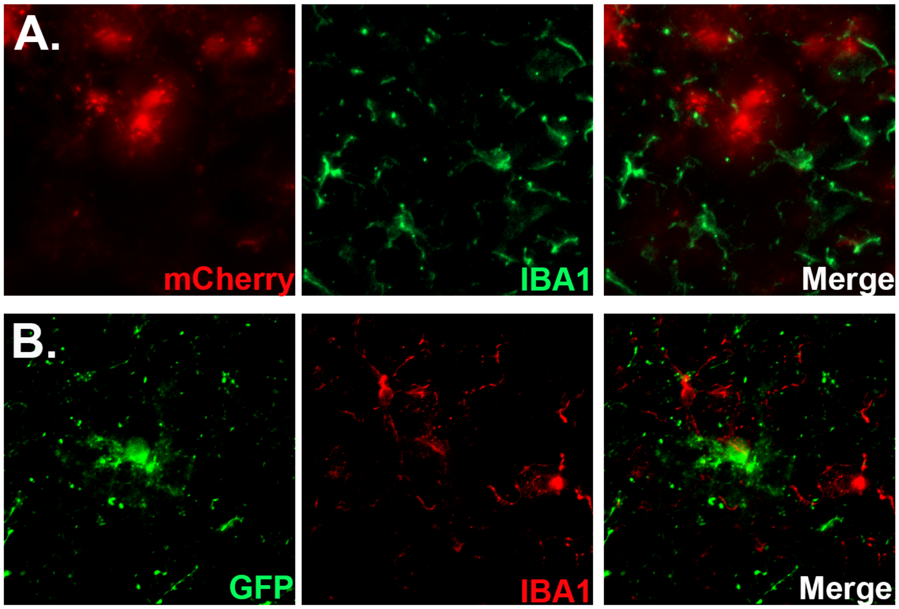

Fig F. AAVDJ8-GFP and AAVDJ8-GFAP-mCherry do not transduce microglia in the SNpc

64X objective immunofluorescent images of (**A**) anti-mCherry (red) and anti-IBA1 (green) depict no co-localization of AAVDJ8-GFAP-mCherry infected cells. (**B**) Anti-GFP (green) and anti-IBA1(red) immunoflourescent images also depict no co-localization of AAVDJ8-GFP (Images are representation of *n*=3/serotype).
